# Insulin resistance in NSCLC: unraveling the link between development, diagnosis, and treatment

**DOI:** 10.3389/fendo.2024.1328960

**Published:** 2024-02-20

**Authors:** Shizhang Zhan, Liu Wang, Wenping Wang, Ruoran Li

**Affiliations:** ^1^ Department of Graduate School, Bengbu Medical College, Bengbu, China; ^2^ Department of Respiratory and Critical Care, Xuzhou Central Hospital, Xuzhou, China

**Keywords:** NSCLC, insulin resistance, development, diagnosis, treatment

## Abstract

Lung cancer is responsible for the highest number of cancer-related deaths, with non-small cell lung cancer (NSCLC) being the most prevalent subtype. A critical aspect of managing lung cancer is reducing morbidity and mortality rates among NSCLC patients. Identifying high-risk factors for lung cancer and facilitating early diagnosis are invaluable in achieving this objective. Recent research has highlighted the association between insulin resistance and the development of NSCLC, further emphasizing its significance in the context of lung cancer. It has been discovered that improving insulin resistance can potentially inhibit the progression of lung cancer. Consequently, this paper aims to delve into the occurrence of insulin resistance, the mechanisms underlying its involvement in lung cancer development, as well as its potential value in predicting, assessing, and treating lung cancer.

## 1 Introduction

Based on the fact that early symptoms of lung cancer are atypical, leading to late-stage diagnosis for the majority of patients, there is a missed opportunity for surgical treatment (1). Although individualized, biomarker-driven therapy, screening for high-risk groups, and surgical treatment have improved survival rates, the results remain unsatisfactory (2). Therefore, it is imperative to identify and screen high-risk groups in order to improve the mortality rate of lung cancer. Current recognized risk factors for lung cancer include smoking, passive smoking, history of chronic obstructive pulmonary disease (COPD), occupational exposure, and genetic susceptibility (1). Recent research has also indicated that insulin resistance is a contributing factor in various cancers (3, 4). While there is growing evidence linking insulin resistance to non-small cell lung cancer (NSCLC), there is currently no clinically accepted threshold to assess insulin resistance (5, 6). Insulin resistance promotes tumor cell proliferation, invasion, and metastasis through mechanisms affecting insulin-like growth factor (IGF)-related pathways, inflammatory response, tumor microenvironment, and reactive oxygen species (ROS) (7–9). Pharmacological interventions, lifestyle changes, and weight loss are believed to reduce insulin resistance and decrease the risk of lung cancer development (10–12). Furthermore, targeted therapy utilizing insulin resistance-related signaling pathways may improve insulin resistance and inhibit the growth and invasion of lung cancer cells (13). In summary, this paper aims to explore the mechanism of insulin resistance in lung cancer, provide an overview of research progress, and deepen understanding of the relationship between insulin resistance and lung cancer to guide the development of new prediction tools and treatment strategies.

## 2 Insulin resistance

### 2.1 Concept of insulin resistance

Insulin resistance, initially proposed by Harold Himsworth in the early 1930s (14), is a metabolic disorder closely associated with obesity and type 2 diabetes. It is characterized by a diminished cellular response to insulin, leading to a reduction in insulin bioactivity (15). This condition has significant implications for overall metabolic health and contributes to the development of various diseases.

### 2.2 Mechanisms of insulin resistance

#### 2.2.1 Abnormal insulin receptor signaling pathway

The insulin receptor serves as the central receptor responsible for insulin’s actions, and any abnormalities in its signaling pathway can result in impaired insulin signaling (16). Evidence suggests that both the expression level and function of the insulin receptor are closely linked to insulin resistance (17, 18). For instance, mutations in the insulin receptor gene can diminish receptor activity, leading to disruptions in insulin binding and subsequent signaling events (19).

#### 2.2.2 Inflammatory response of adipose tissue

Adipose tissue plays a crucial role in the development of insulin resistance. Excessive accumulation of adipose tissue triggers an inflammatory response in adipocytes, which in turn results in the production of various inflammatory factors (20). These factors disrupt insulin signaling pathways and contribute to the onset of insulin resistance. Examples of such inflammatory factors include tumor necrosis factor-α (TNF-α) and interleukin-6 (IL-6) (21).

#### 2.2.3 Abnormal glucose metabolism

Insulin resistance is characterized by a diminished ability of cells to respond to insulin signaling, resulting in impaired glucose utilization and disrupted glucose metabolism (22). As a consequence, glucose is unable to enter cells efficiently, leading to elevated blood glucose levels (23, 24). The increased blood glucose levels, in turn, stimulate the secretion of insulin, establishing a detrimental cycle of insulin and glucose interplay (25, 26). To break this cycle, it is crucial to address the underlying insulin resistance and restore proper glucose metabolism.

#### 2.2.4 Abnormal fatty acid metabolism

Insulin resistance has been shown to have significant effects on fatty acid metabolism (27, 28). It disrupts the balance of fatty acid synthesis, storage, oxidation, and utilization. In the presence of insulin resistance, there is an increased synthesis and storage of fatty acids in adipose tissue. Simultaneously, fatty acid oxidation and utilization are inhibited, causing an accumulation of fatty acids in non-adipose tissues. This abnormal accumulation contributes to the development of disorders in fatty acid metabolism and further exacerbates insulin resistance (29, 30).

#### 2.2.5 Activation of inflammatory response

Insulin resistance often triggers a chronic state of low-grade inflammation in the body (31, 32). The activation of inflammatory response due to insulin resistance results in the infiltration of inflammatory cells and the release of inflammatory factors. These factors can disrupt insulin signaling pathways, further aggravating the condition of insulin resistance (33, 34).

### 2.3 Indicators for assessing insulin resistance

Early and timely recognition of insulin resistance is crucial in preventing the development of related diseases. Clinically, insulin resistance indexes such as the normoglycemic-hyperinsulinic clamp, insulin resistance index (HOMA-IR), and triglyceride glucose index (TyG) are used for assessment. Initially, the normoglycemic-hyperinsulinemic clamp was considered the gold standard for assessing insulin resistance. However, its high cost and complexity limit its use in monitoring the general population (35). As a result, HOMA-IR became a commonly used method to assess insulin resistance, as it does not require routine measurement of insulin levels in clinical settings. However, HOMA-IR has certain limitations (36). In recent years, the TyG index has gained popularity due to its reliability and simplicity and has started to replace HOMA-IR in assessing insulin resistance (37). Studies have shown that the TyG index has a higher predictive value for insulin resistance than HOMA-IR (38). Additionally, a triglyceride glucose body mass index (TyG-BMI) based on the TyG index has been proposed as an alternative index for assessing insulin resistance (6). However, there is still a lack of specific cut-off values to predict insulin resistance, which requires further investigation.

## 3 Correlation between insulin resistance and NSCLC

### 3.1 Epidemiologic studies

Lung cancer remains the second most common malignant tumor in the world, following breast cancer (39). Over the years, numerous studies have investigated the association between insulin resistance and NSCLC by examining a large number of cases. A cross-sectional survey of the Chinese population conducted by Xin Yan et al. found that the TyG index was independently associated with the risk of NSCLC (5). Similarly, Feifei Wang et al. utilized the TyG-BMI index to assess insulin resistance and found it to be a risk factor for NSCLC in the Chinese population (6). Additionally, their study demonstrated that the BMI index was also a risk factor for NSCLC (6). Another prospective study involving 29,133 Finnish men showed that elevated levels of insulin resistance were linked to an increased risk of lung cancer (40). Similarly, Petridou et al. conducted a study on an Athenian population and concluded that insulin resistance was an independent risk factor for NSCLC (41). However, some studies provide contradictory findings. A prospective study conducted by Lijie Wang et al. on a British population showed no significant correlation between the TyG index and the risk of NSCLC (42). These conflicting results may be attributed to differences in lifestyle, dietary habits, geography, and genetic factors among different races. Regardless of insulin intake or impaired insulin utilization in insulin-resistant states, elevated insulin levels may increase the risk of NSCLC. A study on diabetes mellitus and lung cancer in postmenopausal women, conducted by Juhua Luo et al., reported a significant increase in the risk of lung cancer among postmenopausal women with diabetes mellitus after insulin treatment (43). Additionally, Ren Qiu et al.’s study on the correlation between insulin and the non-small cell lung cancer cell line (A549) found that insulin could contribute to the proliferation of lung cancer cells (44). This suggests that, in the state of insulin resistance, excessive insulin accumulates extracellularly and promotes the development of lung cancer. In conclusion, a substantial body of research supports the association between insulin resistance and the development of NSCLC.

### 3.2 Molecular mechanism studies

#### 3.2.1 IGF and the IGF axis

**Figure 1:**
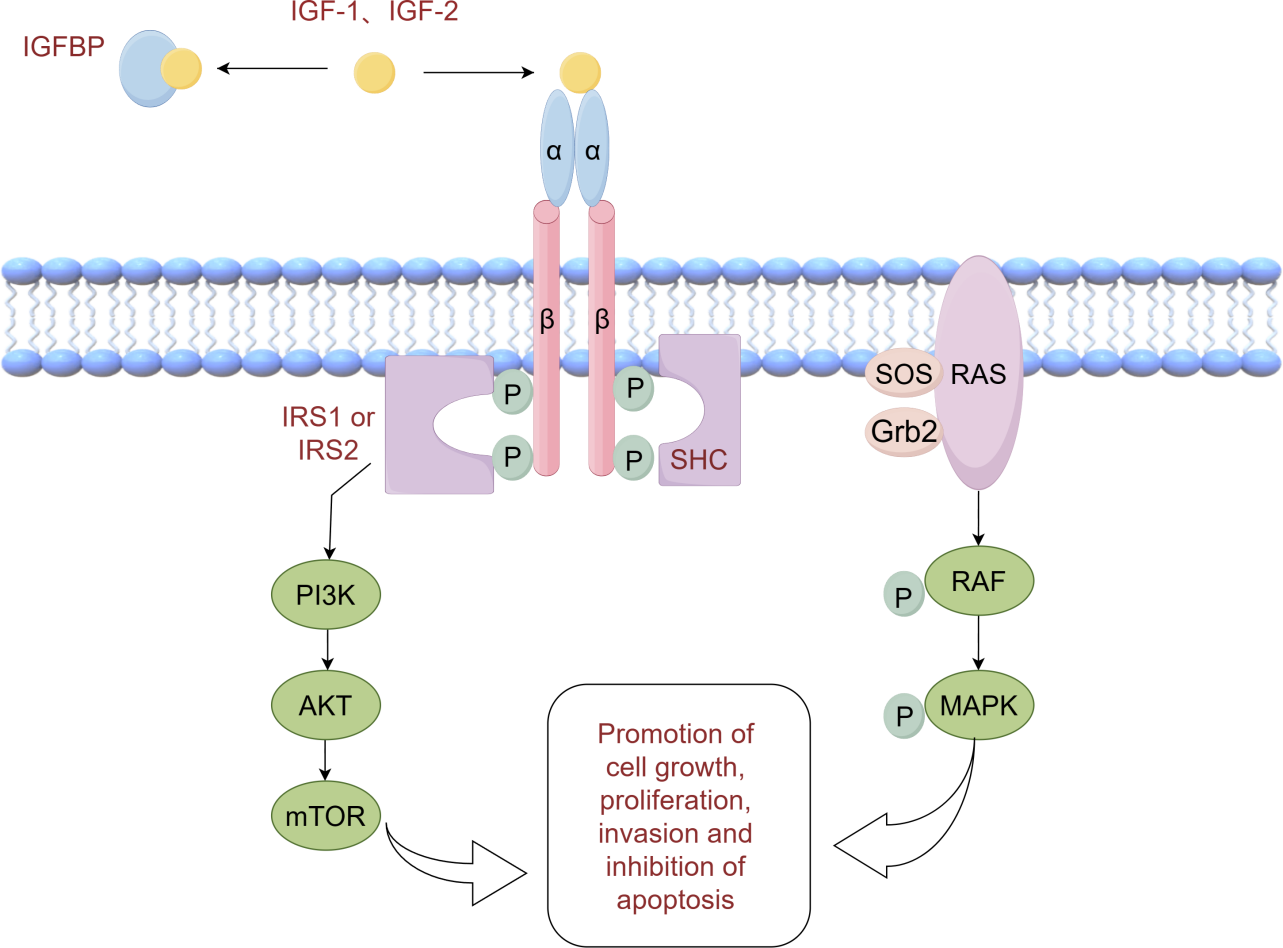
Pathway diagram of IGF (This image was drawn by Figdraw).

Insulin resistance is a condition characterized by reduced biological activity of insulin, leading to an increase in insulin secretion to compensate for its diminished function. However, this increased insulin secretion is often accompanied by elevated secretion of insulin-like growth factors (IGFs) (45). IGFs, which include IGF-1 and IGF-2, are a group of peptide hormones that can promote various cellular processes such as cell proliferation, migration, invasion, and epithelial-mesenchymal transition (EMT). This promotion is achieved through the activation of the phosphatidylinositol-3 kinase (PI3K) and mitogen-activated protein kinase (MAPK) pathways. Structurally similar to insulin, IGF-1 and IGF-2 can bind to both the insulin receptor and the IGF-1 receptor. The binding of IGF-1 and IGF-2 to the IGF-1 receptor induces phosphorylation of the receptor itself, which in turn phosphorylates its binding proteins, including insulin receptor substrate-1 (IRS-1) and Src homologous collagen (SHC). This phosphorylation activates the PI3K/Akt and Ras/MAPK signaling pathways, respectively, resulting in enhanced cell growth, proliferation, invasion, and inhibition of apoptosis (46, 47). **Figure 1** illustrates the molecular mechanisms involved. Numerous studies have demonstrated increased expression levels of IGF-1 and IGF-2 in NSCLC tumor tissues (48, 49). Interestingly, a study by Kim et al. discovered that low expression of IGF-1 was associated with longer overall survival (OS) in NSCLC patients. This finding emphasizes the significance of IGF-1 as a prognostic indicator and suggests the potential for risk stratification in NSCLC based on IGF-1 levels (50).

The IGF axis plays a crucial role in the growth, invasion, and metastasis of NSCLC, with molecular proteins such as IGF-1R, IGF-2R, and insulin-like growth factor binding protein (IGFBP) being implicated (51). The expression of IGF-1R in NSCLC tissues is closely associated with that of IGF-1 and serves as an independent negative predictor of NSCLC prognosis (49, 52). This negative effect may be linked to the co-expression of IL-6 and EGFR (53). A meta-analysis revealed that high expression of IGF-1R predicts a lower likelihood of disease-free survival (DFS) in NSCLC patients (54). Moreover, elevated levels of IGF-1 have been found to be a negative prognostic indicator and associated with poor survival in NSCLC patients (55). Consequently, targeting IGF-1R shows promise as a therapeutic approach and prognostic biomarker for NSCLC. Similarly, low expression of IGF-2R is associated with a worse prognosis in advanced NSCLC (56). Additionally, IGFBP competitively binds to IGF, preventing it from binding to the IGF receptor, and demonstrates anti-metastatic, anti-angiogenic, and anti-tumor activity in NSCLC cells (57). Several studies have explored intervention strategies targeting the IGF axis for lung cancer progression. For instance, IGF-1R inhibitors have been investigated for lung cancer treatment, blocking the activation of the IGF-1R signaling pathway and inhibiting the proliferation and invasion of lung cancer cells (58, 59). In addition, modulation of IGF activity by regulating the level of IGFBP has been explored (60, 61).

#### 3.2.2 The tumor microenvironment

Insulin resistance has a significant impact not only on the biological properties of tumor cells but also on the tumor microenvironment, thereby promoting the development of lung cancer (62). In the state of insulin resistance, there are aberrations in the insulin receptor signaling pathway, specifically a reduction in tyrosine phosphorylation of IRS1 and inhibition of the PI3K/Akt signaling pathway (63). Consequently, cellular response to insulin is compromised, affecting glucose metabolism and cell proliferation. Due to the inability of cells to effectively utilize glucose as an energy source, they increase their demand for alternative nutrients, such as fatty acids and amino acids, to compensate for the lack of energy. This metabolic shift creates a more favorable environment for tumor cell survival, growth, and metastasis (64, 65). Furthermore, it has been observed that insulin-resistant tumor tissues exhibit enhanced inflammatory responses and increased secretion of inflammatory factors, such as TNF-α and interleukin-6 (IL-6) (66). These inflammatory factors stimulate the production of growth factors and angiogenic factors within tumor cells, further facilitating tumor growth, invasion, and metastasis (67).

#### 3.2.3 Inflammatory response and immune escape

Enhanced inflammatory responses in insulin-resistant states have been associated with the progression of lung cancer. It has been observed that insulin resistance can stimulate the production and release of inflammatory factors by activating inflammation-related signaling pathways, such as the NF-κB-related pathway. These inflammatory factors contribute to the promotion of tumor cell growth, invasion, and metastasis (8, 68). Moreover, insulin resistance is also implicated in the progression of lung cancer through its influence on tumor immune escape. In insulin-resistant states, T cell function is suppressed, leading to a reduced ability to recognize and eliminate tumor cells. Consequently, this creates a favorable environment for tumor cell growth and metastasis (9, 69). Additionally, insulin resistance can alter the interaction between tumor cells and the surrounding stroma by affecting the expression of cell adhesion molecules and matrix metalloproteinases. This, in turn, facilitates cell invasion and metastasis (70).

#### 3.2.4 Reactive Oxygen Species (ROS)

Insulin resistance is known to be associated with the increased production of ROS, although the exact mechanisms underlying this association are not yet fully understood. Current research suggests several potential pathways through which insulin resistance can lead to ROS production. One possible mechanism is the impairment of mitochondrial function within the cell. Mitochondria are responsible for energy production, and when their function is compromised, they may generate more ROS as a byproduct of the oxidative phosphorylation process (71). Hence, insulin resistance may contribute to mitochondrial dysfunction, resulting in elevated ROS production. Another factor linking insulin resistance to ROS production is chronic low-grade inflammation. Research indicates that insulin resistance can promote inflammatory responses, which, in turn, can increase the production of ROS (72). The activation of inflammatory pathways due to insulin resistance may contribute to an amplified intracellular inflammatory response, leading to higher levels of ROS. Additionally, insulin resistance has been found to increase the activity of an enzyme system known as NADPH oxidase. This enzyme system is responsible for reducing oxygen to reactive oxygen species, thereby generating ROS within the cell (73). When NADPH oxidase activity is elevated, there is a corresponding increase in ROS production. The excess production of ROS overwhelms the cell’s antioxidant defense mechanisms, ultimately resulting in cellular damage. It has been proposed that ROS can induce DNA damage, gene mutations, and apoptosis, which are all significant factors in the development of cancer (74). Furthermore, there is evidence suggesting that ROS can impact the progression and prognosis of lung cancer (75).

## 4 Regulation of insulin resistance and lung cancer

### 4.1 Dietary regulation

Several studies have demonstrated that dietary factors can impact the association between insulin resistance and lung cancer. For instance, incorporating high-fiber diets, reducing saturated fats, and consuming foods rich in antioxidants may potentially enhance insulin resistance and lower the risk of developing lung cancer (10, 11). Moreover, natural products like caffeine and tea polyphenols have been indicated to ameliorate insulin resistance and inhibit the progression of lung cancer (76). These findings highlight the potential benefits of dietary modifications and the use of natural compounds in reducing the risk of lung cancer by improving insulin resistance.

### 4.2 Physical exercise and weight management

Physical activity and weight management play crucial roles in regulating insulin resistance and reducing the risk of lung cancer. Research has demonstrated that engaging in moderate exercise can enhance insulin sensitivity, lower insulin resistance, and subsequently decrease the likelihood of developing lung cancer (77). Moreover, maintaining a healthy weight is particularly vital in combating insulin resistance since obesity is closely associated with this condition. By achieving and sustaining a healthy weight, individuals can effectively mitigate the risk of both insulin resistance and lung cancer (78).

### 4.3 Pharmacologic interventions

Numerous studies have investigated the potential of pharmacological interventions in improving insulin resistance and preventing lung cancer development. Metformin, an insulin sensitizer, has been extensively studied for its ability to inhibit the proliferation and invasion of lung cancer cells by enhancing insulin resistance and influencing metabolic pathways (12). Molecularly targeted therapies have also been explored to modulate insulin resistance and impede lung cancer progression. For instance, targeting the PI3K/Akt/mTOR signaling pathway is believed to ameliorate insulin resistance, thus inhibiting the proliferation and invasion of lung cancer cells (13, 79). Furthermore, other targeted drugs, such as IGF-1R inhibitors and AMPK activators, have demonstrated potential in modulating insulin resistance and lung cancer (80, 81).

## 5 Summary and clinical outlook

**Figure 2:**
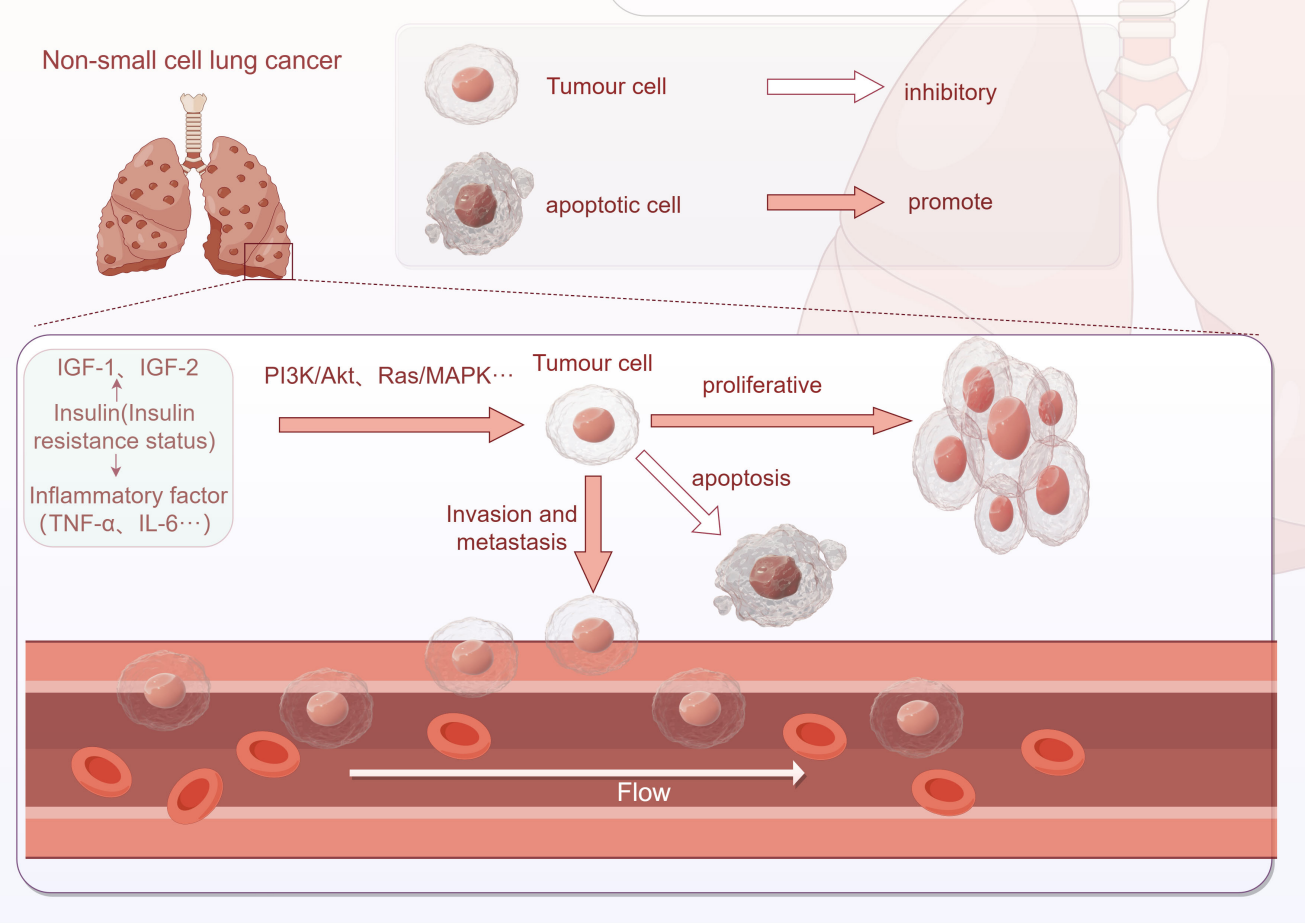
Association between insulin resistance and NSCLC development (This image was drawn by Figdraw).

There is a strong connection between insulin resistance and the development of lung cancer. In individuals with insulin resistance, elevated insulin levels play a significant role in the initiation and progression of lung cancer. This association between insulin resistance and lung cancer involves various pathways. Firstly, insulin resistance promotes the proliferation, invasion, and metastasis of tumor cells. Additionally, insulin resistance activates inflammatory responses, inhibits apoptosis, and impacts immune escape mechanisms. These findings are illustrated in **Figure 2**.

Insulin resistance has emerged as a potential therapeutic target in the clinical setting, particularly for the treatment of lung cancer. Numerous studies have explored the use of insulin resistance modulators to intervene in the development of this disease. Specifically, insulin-sensitizing agents and inhibitors of insulin-like growth factor-1 receptor (IGF-1R) have been investigated as potential treatments for lung cancer, aiming to inhibit aberrant signaling and cell proliferation associated with insulin resistance. Furthermore, drugs that can modulate insulin resistance, such as insulin sensitizers and anti-inflammatory agents, may also hold promise in the treatment of lung cancer.

However, there are still numerous areas that remain uncharted and require further research regarding the relationship between insulin resistance and lung cancer. Firstly, the accurate assessment of the extent of insulin resistance needs to be addressed. Secondly, the causal relationship between insulin resistance and lung cancer needs to be explored in depth. Finally, it is crucial to investigate the molecular mechanisms underlying insulin resistance. In order to gain a better understanding of these aspects, more comprehensive clinical studies are warranted. These studies will play a vital role in validating the efficacy and safety of insulin resistance modulators for the treatment of lung cancer.

A comprehensive investigation into the connection between insulin resistance and lung cancer can enhance our understanding of the mechanisms involved in the development of lung cancer. This study can also provide a solid foundation for the development of targeted therapeutic strategies. By effectively regulating insulin resistance, we aim to offer improved treatment options for lung cancer patients, ultimately leading to enhanced survival rates and improved quality of life.

## Author contributions

SZ: Conceptualization, Writing – original draft. LW: Resources, Writing – original draft. WW: Project administration, Writing – original draft. RL: Conceptualization, Writing – review & editing.
